# Implementing Case Management with Positive Youth Development to Empower Young Mothers in California

**DOI:** 10.1007/s10995-020-02985-5

**Published:** 2020-08-03

**Authors:** Subuhi Asheer, Betsy Keating, Jacqueline Crowley, Susan Zief

**Affiliations:** Mathematica, P.O. Box 2393, Princeton, NJ 08543 USA

**Keywords:** Case management services, Expectant and parenting youth, Positive youth development, Implementation study

## Abstract

**Objectives:**

With funding from the Pregnancy Assistance Fund, the Maternal, Child, and Adolescent Health Division (MCAH) of California redesigned its existing Adolescent Family Life Program (AFLP) for expectant and parenting young women into a more intensive and structured intervention, AFLP with positive youth development (PYD). This paper presents key findings from a federally funded, rigorous implementation study of the two programs.

**Methods:**

This implementation study collected data from 13 agencies from January 2016 through December 2017, including interviews with 69 case managers and 18 supervisors; focus groups with 130 program participants; surveys of 66 case managers and 1330 young women; and observations of 42 visits with program participants. The study combined qualitative and quantitative analysis methods.

**Results:**

As designed, PYD was a much more structured and intensive program than AFLP. Case managers and supervisors saw value in the PYD model and new approach but needed more support and guidance than expected in order to deliver it with fidelity. MCAH provided additional trainings and technical assistance to address challenges. In practice, although staff noted differences in approach and content, the youth experience with the two programs was similar.

**Conclusions for Practice:**

Integrating the PYD framework into case management systems may foster youth self-sufficiency and resiliency. However, the rigid structure of the program was often challenging to implement in practice. Organizations interested in implementing prescribed case management approaches should consider allowing opportunities for flexibility in implementation and providing more detailed preservice training to prepare staff for real-world implementation.

## Significance

### What is Already Known on This Subject

The positive youth development framework is an evidence-informed approach for improving adolescents’ outcomes, but little is known about its effectiveness in case management with young parents on a large scale. California’s Adolescent Family Life Program (AFLP) had served expectant and parenting youth for about two decades, although the quality, content, and structure of implementation varied considerably.

### What this Study Adds

This paper examines the implementation of California’s newly redesigned case management program compared with the existing AFLP. Informed by multiple data sources, this statewide implementation study offers lessons for practitioners and researchers interested in case management using a systematic positive youth development approach to meet the needs of young parents in diverse communities.

## Introduction

Among programs serving adolescents, the last few decades have seen a shift toward using an evidence-informed positive youth development (PYD) approach to promote better health and education outcomes (Catalano et al. 2004; Gloppen et al. 2009; Lerner and Lerner 2013). The PYD framework sees adolescents as active partners—who bring their own voice, values, and resources for defining a path to success—rather than as passive program recipients with problems that need fixing (Zarrett and Lerner 2008). Critical elements of the PYD approach include building competence for independent decision making, developing adolescents’ confidence through skill building, identifying and using their strengths and values to set and meet specific goals, and encouraging self-care and self-advocacy (Zarrett and Lerner 2008). Using the PYD framework to help expecting and parenting adolescents may nurture and strengthen protective factors, reduce risky behaviors and the chances of a rapid repeat pregnancy, and foster academic and social success in the long term (Catalano et al. 2004).

For more than a decade, the California Maternal, Child, and Adolescent Health (MCAH) division had administered the Adolescent Family Life Program (AFLP), a case management program for expectant and parenting youth. Using a grant from the Pregnancy Assistance Fund (PAF) in 2010, MCAH integrated a PYD approach into a new version of its AFLP program in a select group of sites (the original AFLP program continued to operate in the state at the same time). Drawn from evidence-informed positive youth development principles (Catalano et al. 2004; Gloppen et al. 2009; Lerner and Lerner 2013), the new program (1) prescribed a set of structured activities and content to help youth identify their strengths and use them meet their goals and (2) required that case managers conduct two visits a month instead of one. Pressfield, Campa, Ramstrom, Kabadi, and Lopez in this supplement provide more details on the development of the new program model (referred to as PYD in this paper).

**Fig. 1:**
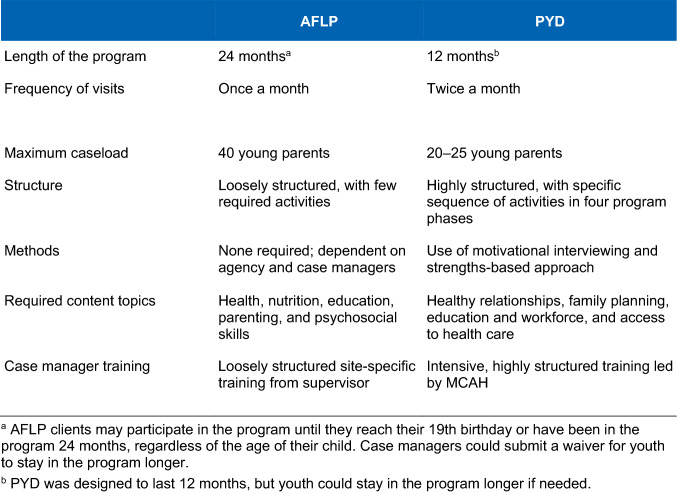
A comparison of key features of AFLP and PYD

**Fig. 2:**
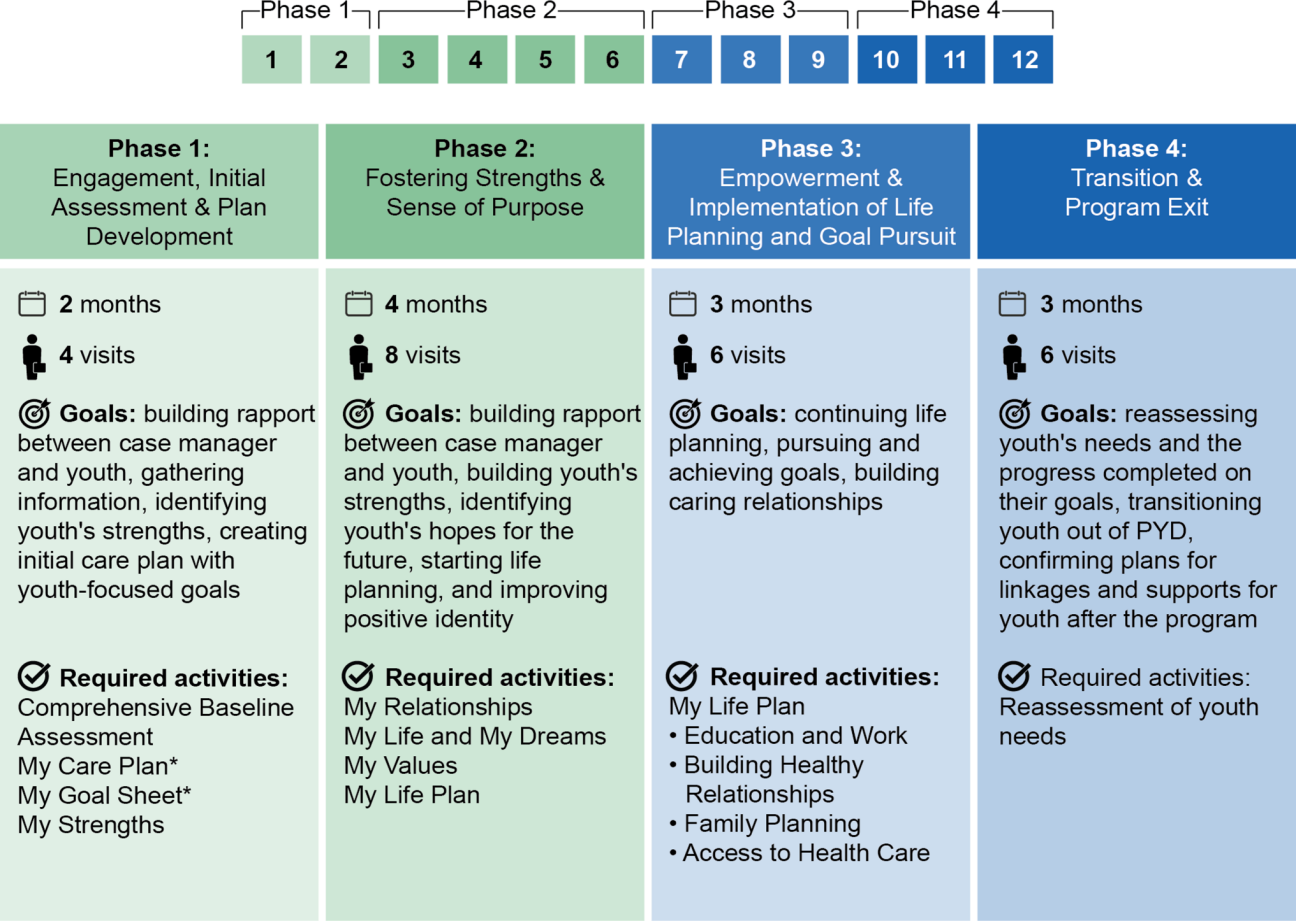
PYD phases: goals and required activities

As designed, PYD reflects a clear contrast with the AFLP program in terms of the approach, methods, and structure (Fig. 1). PYD is a shorter but more intensive program than is AFLP. PYD case managers meet with clients two times a month for one year and have caseloads of 20 to 25 youth, compared to monthly visits for two years and caseloads of 40 youth for AFLP case managers. PYD requires that case managers use motivational interviewing to guide participating women through a prescribed set of activities designed to help them set achievable goals for life planning and build self-sufficiency (Fig. 2). The content and activities, organized in four phases, utilize a strengths-based approach to encourage the young women to define and build on their strengths and successes. MCAH requires PYD case managers to complete MCAH-led trainings on the program and monthly technical assistance (TA) calls, whereas AFLP case managers complete site-based trainings that can vary in intensity and methods based on site’s requirements.

**Fig. 3:**
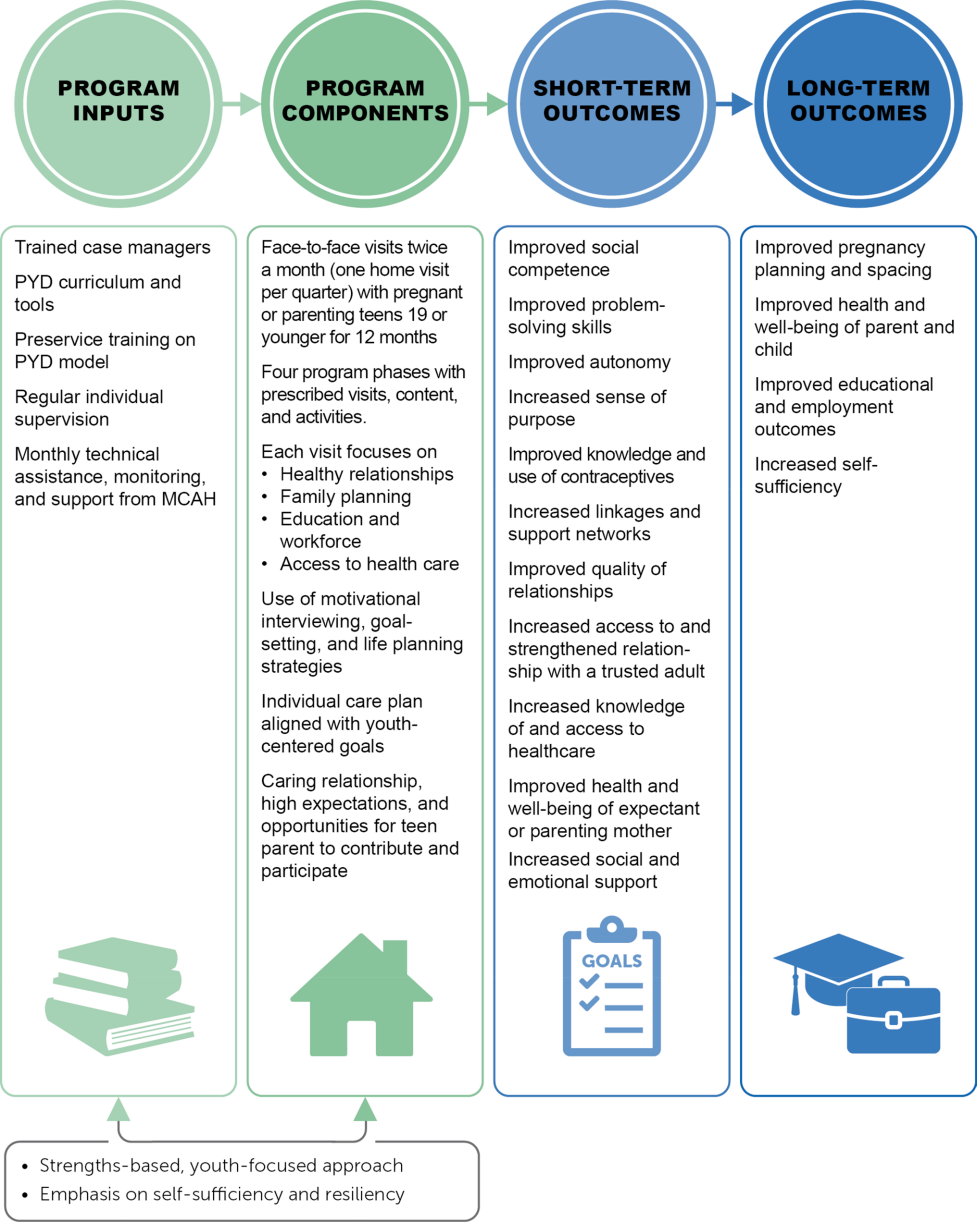
PYD logic model

Like AFLP, PYD seeks to delay or prevent repeat pregnancies, increase high school completion, and improve the health of the parent and child. In the short term, PYD adds a more explicit focus on improving social competence, problem-solving skills, autonomy, sense of purpose, and relationship quality (Fig. 3). In the long term, the program is also designed to increase the participant’s self-sufficiency and improve linkages to services, community, and support networks that the participant can lean on as she transitions out of the program and becomes more self-reliant.

This paper examines the implementation of two programs in California during a randomized controlled trial evaluation. A separate forthcoming publication from the Office of Population Affairs (OPA) will provide details about the study design and share one-year program impact results, and a later publication will include the two-year program impact results (Dragoset et al 2020). The current study focuses on the implementation of AFLP and the recently redesigned PYD from December 2014 to February 2017, during which sites were randomly assigned to deliver either AFLP or PYD.^1^ The paper explores the following key research questions:

- How were staff delivering PYD prepared and supported, compared to those delivering AFLP?
- How was PYD implemented on the ground, relative to what was intended and compared to AFLP?
- How did the experiences of staff and youth participating in PYD differ from those in AFLP?

Findings from this study provide important lessons for practitioners and researchers interested in refining or enhancing case management models for young parents using a prescribed positive youth development approach and structure, along with more frequent contact with case managers. The paper describes necessary and important considerations for practitioners implementing this more intensive PYD case management model with young women, along with specific successes and challenges related to that process.

## Methods

The AFLP and PYD evaluation, which included both an impact and implementation study, was funded by OPA (formerly Office of Adolescent Health) and conducted by Mathematica. Thirteen sites and 1330 youth participated in the evaluation and completed one baseline and two follow-up surveys (at 12 and 24 months after the program). 2,3 Based on data from the youth baseline survey, the participating young women were primarily Hispanic, 16 to 18 years old at the start of the study, and had one child. Nearly all did not yet have a high school diploma or equivalent, and most (80%) were still enrolled in school. The case managers delivering the two programs were similar: most were Hispanic, held a bachelor’s or master’s degree, and were 40 years old on average. They also had comparable prior work experience, with about two-thirds of case managers in both programs having served teen parents for five years or more prior to the study, including teens who had participated in AFLP.

**Fig. 4:**
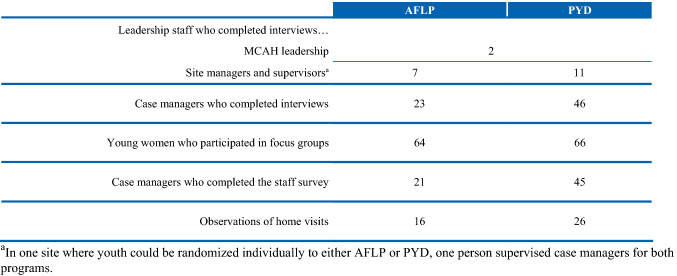
Sources of data for the implementation study of AFLP and PYD

The first author and another member of the implementation study team collected data in 2016 and 2017 from several sources and engaged with multiple respondents across the participating sites to ensure that a variety of perspectives were included (Fig. 4).^4^ Case managers completed a staff survey about a year after implementation began, providing information on their background, training, and experiences with the programs. 5,6 To better understand staff experiences with program implementation, the team conducted semi-structured 60-min in-person interviews with case managers and their supervisors who oversaw the day-to-day operations at each site, and with MCAH program administrators.^7^ The team interviewed all supervisors and case managers working with AFLP or PYD in participating sites at the time of the interviews; staff at each site helped coordinate the scheduling of interviews. In addition, the team conducted 20 in-person, 90-min focus groups with AFLP and PYD participants across all sites.^8^ The team used a semi-structured protocol to guide the discussion during the focus groups. Case managers helped recruit participants for the focus groups by inviting all participants to attend either during their visits or via text or phone. No one besides the respondents and researchers was present during the focus groups or staff interviews. The team recorded the interviews and focus groups and later completed verbatim transcriptions. The study team conducted a small number of observations of AFLP and PYD visits to better understand the women’s engagement, the case managers’ facilitation practices, time allotted to specific topics, and the overall quality of visits. Observations were a convenience sample based on the timing of visits at each site; the observations may not be representative of all AFLP and PYD visits. The team also examined aggregate summary data that MCAH provided on average monthly caseloads across case managers participating in the evaluation and average number of visits across all youth enrolled in the evaluation over the first two years of program implementation. Finally, the study drew on data from the youth baseline survey to assess background characteristics. All data were collected in accord with prevailing ethical principles and reviewed by an Institutional Review Board. This manuscript is not based upon clinical study or patient data. The study team followed the COREQ criteria for reporting qualitative research (Tong et al. 2007).

The study combined qualitative and quantitative analysis methods. For the interviews and focus groups, the team first developed a structured coding scheme to organize data around the sections of the semi-structured protocols used for the interviews and focus groups, so as to capture information related to the research questions. After developing a detailed codebook outlining the definition of each code, the team trained three staff members on the coding scheme and codebook. These three individuals then coded the transcripts in NVivo. Throughout the coding process, more-senior members of the team conducted quality assurance reviews of selected coded transcripts and provided feedback to the coders as needed to ensure all team members applied the coding scheme reliably and consistently. After coding was completed, the team used results queries from NVivo to systematically analyze the data and identify key themes. The observation data had open-ended responses that were considered alongside the qualitative queries from NVivo and had quantitative data through numerical quality ratings and the percentage of time in each session spent on particular topics. The team averaged the quantitative observation data across all observations for each program. The staff and youth baseline survey data were analyzed in SPSS and Stata, using unweighted tabulations. The team presented findings from all data sources to MCAH program administrators and requested their feedback.

## Results

In redesigning AFLP to create PYD, MCAH shifted the program toward a holistic, youth-centered model that emphasized strengths and values and focused on building self-sufficiency. Case managers and supervisors liked the foundational principles, approach, and content of the program but found that implementing this intensive and structured model as intended required more time and support than the state and sites initially expected.

### Preservice Training for PYD Was Intensive But Did Not Fully Address Early Case Manager Needs

Staff in the two programs had very different experiences with training. As expected, staff preparation for AFLP was implemented at the site level, so it was largely driven by individual site policies and procedures. The materials covered and guidance on methodology or approach varied considerably across sites. At a minimum, most new AFLP case managers reviewed the program and administrative requirements with their supervisors (such as the core topics to be covered in visits, local resources and referrals, and so on); often shadowed more experienced case managers for one to two weeks; and reviewed site-specific manuals. Although AFLP training was not standardized across sites, the training was based on long-established procedures developed through years of implementing AFLP at each site. AFLP staff were also expected to adhere to state-mandated reporting and monitoring procedures, such as documentation of visits and monthly caseloads.

In contrast, the training experience for PYD staff was more intensive than for AFLP and covered a lot of ground. PYD case managers and supervisors all attended a highly structured, two- to four-day in-person preservice training.^9^ Conducted by two MCAH staff, the training incorporated discussions on the theoretical framework of the new PYD model and explained the phased program structure and the required activities for each phase. Staff also provided guidance on completing paperwork for the new PYD activities. The training primarily relied on lectures with some small and large group activities, videos, and worksheets. Following the preservice training, MCAH held monthly TA calls with PYD supervisors. These calls discussed progress toward key program expectations through two key metrics for case managers: the number of monthly visits per client and the number of women on case managers’ caseloads; they also provided opportunities for supervisors to share specific challenges or questions. Despite the increased support from MCAH, though, PYD case managers did not have an opportunity to shadow more seasoned case managers to learn and observe in the field because the program was new. AFLP case managers had explained that shadowing was an important aspect of training and improving staff comfort with the model, and PYD case managers noted this gap in their training.

Through these early conversations with sites, it became clear that supervisors and case managers were struggling to implement PYD after the initial training. The preservice training gave staff a good foundation for the new model’s approach and content. However, supervisors and case managers emphasized that the program felt “inflexible” and they needed more guidance on how to deliver it day to day as planned, especially with women who were facing crisis. Supervisors also indicated that they needed more specific guidance for their role in developing appropriate systems of support and oversight for their case managers.

**Fig. 5:**
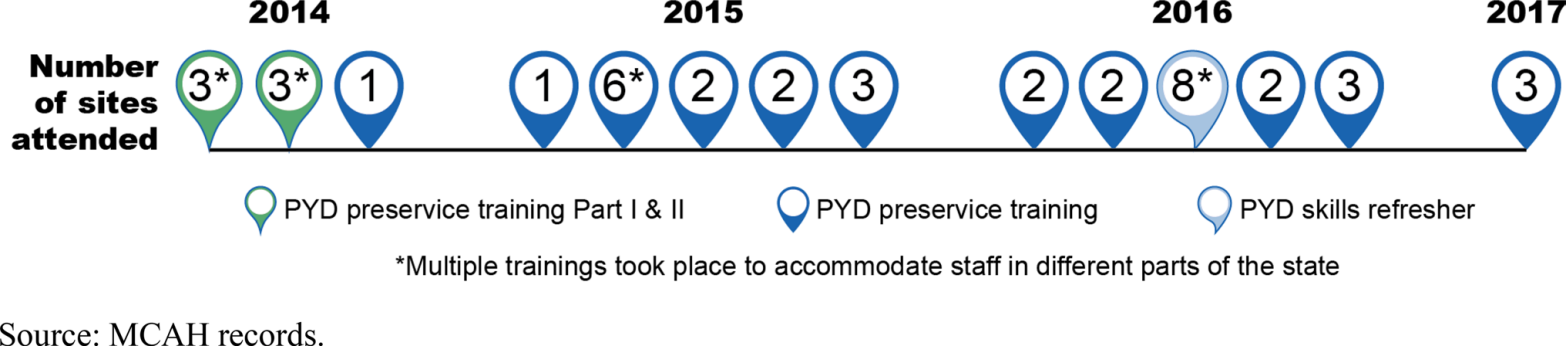
Timeline and number of PYD trainings offered by MCAH

To address the limitations of the initial PYD training, MCAH staff identified further opportunities for training and targeted support across all sites. Beginning about six months after the start of program delivery, most supervisors and case managers attended one or more two-day training(s) led by MCAH and designed to build on the preservice training (Fig. 5); address practical questions about implementing the model, including how to move through each of the four PYD phases; and to help align site-specific systems and processes with the new PYD expectations. One MCAH trainer noted, “We want [the case managers] to focus on being purposeful, using emotional regulation, and how you deal with difficult situations. That is an increased focus during the training, and we are continuing to work with case managers to use that approach, even when youth are in crisis.” Supervisors and case managers said they found the additional trainings helpful in improving their understanding of program expectations and approach and wished they had received some of the more tailored guidance sooner. MCAH visited sites that were struggling with implementation to better understand their structure, needs, and concerns and provided targeted guidance on the types of documentation and adjustments needed to meet program expectations. Finally, MCAH staff restructured the order of the conversations on the TA calls to provide more space and time for sites to talk and share and to do more intentional, concrete planning at the site level. Across trainings, site visits, and TA calls, the messaging from MCAH became clearer and more consistent for case managers over time, which helped them feel more confident about delivering PYD as designed.

### Staff Found They Needed a More Intentional Approach to Implement PYD Compared to AFLP

Unlike AFLP, which had been in place for decades, PYD was a new approach for the sites assigned to deliver the program and, as noted by supervisors, required a significant shift in mindset. Both AFLP and PYD emphasize the importance of goal setting, but the approaches are quite different. The AFLP visits typically had free-flowing discussions centered around case managers getting updates on the five core content areas: the young women’s reproductive health and contraceptive status, family relationships and support, progress on educational and career goals, and parenting and child development needs. AFLP case managers explained they prepared for their visits by compiling the resources and referral information they would need for each woman. The types of materials varied by site but generally included common resources such as local job applications, course listings or requirements for applying to community college, referrals to contacts at local alternative high schools, flyers on child development milestones and types of birth control, and more. Although AFLP had no required approach for the program, AFLP case managers highlighted the importance of early trust building and the strategies they used to develop rapport with the women. They also indicated that the women really wanted to talk about their pregnancy or the new baby’s development. As teenagers, they also had many questions or concerns about parenting, fears about what to expect, and few resources to guide them. Case managers said this was an important motivation for the women to participate in the program and helped forge strong bonds and trust in the long term.

In contrast, PYD’s prescribed activities and materials for each visit were designed to support the young women in taking ownership and driving their goal setting and problem solving rather than relying on the case manager. To complete PYD activities as intended, case managers and clients needed to slow down, step back, and be more purposeful in their goal setting. In describing the differences between the approaches, one PYD case manager stated, “With AFLP it was like I was driving the car, and now I can let the client be in the driver’s seat and take ownership for what is going on in their life.” PYD case managers appeared to have incorporated a shift to more youth-driven activities: on the staff survey, nearly twice as many PYD case managers (80%) agreed that youth should take the lead in goal setting, compared with about 40% of AFLP case managers.

As they grew more comfortable with the new model, staff identified the strengths of the PYD model and approach. For instance, staff believed the set of activities associated with the My Life and Me component (such as My Strengths) was particularly useful in supporting the women in building resiliency and overcoming challenges independently. As several case managers noted, most of the young women had never stopped to think about their strengths or their values and how those could be leveraged in their daily lives to solve problems—many clients expressed being pleasantly surprised when they were able to identify multiple strengths that had served them well.

In addition, case managers found it initially challenging to balance meeting the immediate needs of the women in crisis with completing the required PYD activities in each visit. Although many of the young women enrolled in both programs faced significant life challenges and immediate crises, those enrolled in PYD were often not emotionally or physically prepared to participate in all of the prescribed activities, which required thoughtfulness, presence of mind, and a level of stability to discuss the future and set goals. Nearly three-quarters (73%) of PYD case managers reported on the survey that they had to spend visits helping to address clients’ crises, which took time away from covering the program’s topics. In comparison, just 24% of AFLP case managers said that crisis management took away from their time covering the program’s topics. For youth with more immediate needs, PYD case managers found that they needed more flexibility to move through the four PYD phases. Most PYD case managers said they needed additional time and more guidance and support from MCAH to find ways to follow the program while helping the women meet their basic needs in a seamless way.

### In Practice, Visit Frequency Increased Under PYD But Was Not as Intensive as Originally Planned

As expected, PYD case managers had smaller caseloads than AFLP case managers did. Ideally, the reduction was to provide more time for case managers to conduct additional visits with participating women and forge a deeper connection. The staff survey showed most PYD case managers had lower caseloads than did AFLP case managers a year after they began implementing the program. This finding aligns with MCAH administrative records collected from each site that confirm that PYD case managers’ average caseloads decreased over the course of the first two years of implementation from 23 to 19 clients per month, whereas AFLP caseloads were significantly higher, at about 30 clients per month at the end of the two-year period.

However, despite lower caseloads, PYD case managers across all sites said they found it nearly impossible to meet all of their clients twice a month during the study period. Missing appointments and frequent rescheduling were common across both programs but were more pronounced for PYD where twice as many visits needed to be scheduled. In interviews, PYD case managers explained barriers to meeting clients twice a month, including transitions in clients’ lives, transportation issues, family and school commitments, and other overwhelming life circumstances. For many participants, even the one visit could be challenging. Housing instability meant that families moved often, and it took time and effort to locate them again and schedule the visits. Administrative data collected by MCAH show that on average, women enrolled in PYD during the first year that it was delivered received 1.6 visits per month and 1.4 visits per month in the second year of implementation. This is slightly higher than an average of 1.1 visit per month for AFLP clients in the same period but does not meet the two visits a month expectation. Supervisors and case managers indicated that the two-visit approach was not necessary or possible with all young women and that the model could use more flexibility around the number of visits required.

### Youth Experience with PYD and AFLP Was Similar in Quality but Varied in Content

Although both programs are predicated on a strong relationship between the case manager and youth, PYD’s intentional approach and intensive visit structure and frequency were designed to be deeper, strengths based, and more collaborative. From the perspective of the implementation study staff who observed a small number of AFLP and PYD visits,^10^ along with the youth who received the program, the overall quality of visits in both programs was high and similar across the two programs. Women enrolled in both AFLP and PYD appeared highly engaged in the discussions with their case managers. Among the visits observed, case managers in both programs typically used open-ended questions and incorporated planning and goal setting into the conversations. Case managers across the board were also extremely empathetic, patient, and positive in their approach—their interactions showed they clearly cared about their clients.

In focus groups conducted with 130 participants across the two programs, women in both AFLP and PYD spoke highly about their case managers. They felt a close bond and referred to them as their friends or parents. The young women reported that the case managers were instrumental in motivating them when they needed encouragement, understood their challenges and problems, helped them identify and feel positive about their goals, and were there for them when the women needed anything for themselves and their families. Several women also talked about how their case manager was the reason they did not give up on their education and future careers. For example, an AFLP participant shared that before enrolling in the program she did not aspire to “big things,” but as a result of her case manager’s help, she got into college and now plans to be a dental assistant. One PYD participant said her case manager helped her learn how to “face her problems”. Overall, youth in both programs noted that their case managers often filled a void or need in their lives.

Although this study found no apparent differences between the perspectives of women enrolled in AFLP and PYD on their relationships with their case managers, those participating in PYD said they found the program’s content and the strengths-based approach valuable. Most women receiving PYD said that the activities helped them think about their values and strengths in ways they had not considered before. They said they could now better apply their strengths and solve problems in their lives. For example, one mother took her completed My Strengths worksheet with her to a job interview to help her articulate what she would bring to the job. Another shared that she and her case manager looked back at what they had written down about her accomplishments, which helped serve as a reminder of all she had done and motivated her when she felt discouraged.

During visit observations, in addition to quality, the study team also examined the average percentage of time spent on specific topics for both programs. Among the visits observed, AFLP case managers spent more time addressing infant health and development, family planning, and access to health care, whereas PYD case managers spent more time addressing education and employment, providing resources and referrals, and healthy relationships. Observers noted that some of the content varied significantly based on the needs and circumstances of the clients observed, so broad conclusions about the content cannot be drawn based on these data. However, these data appear consistent with the greater focus on building self-sufficiency through future planning and developing a network of resources in the PYD activities and with the relatively smaller number of activities centered on child development compared with AFLP.

## Conclusions for Practice

As designed, a clear contrast between the AFLP and PYD programs exists, but in practice, their difference is more complicated. Starting with a foundational emphasis on positive youth development principles, the new model added prescribed content and methods and a more intensive visit structure to be delivered over one year. PYD case managers received more intensive preservice training and materials to prepare them, compared to AFLP case managers, and had lower caseloads. However, implementing the new, highly structured model as intended required more time and support than the state and sites initially expected. Most staff used and appreciated the new strategies that emphasized women’s strengths and self-sufficiency but thought that integrating flexibility into the program’s design was critical to developing initial rapport and a deeper connection with their clients. They found it challenging to complete the required two visits a month (compared to the one required visit for AFLP) and to integrate new content with meeting young mothers’ immediate needs. The women’s experience with both AFLP and PYD was comparable in quality based on focus group discussions and observations, though the content of visits varied.

This study has a number of limitations. First, the study takes place in the context and environment of one state, so findings may not be applicable to other states or contexts. Given its scope, the implementation study did not collect a representative sample of observation data to be able to systematically and statistically examine and differentiate the facilitation quality of the two programs, given the differences in intended methods and approach. With limited requirements around methods or specific content for each visit, AFLP has more flexibility and was not implemented consistently across sites or among participating women. This reality may complicate the interpretation of comparing the implementation of a program with a highly structured, prescribed approach with one that had none. Finally, the study focused on program implementation to expectant and parenting women enrolled in the two programs. Important lines for further inquiry include an examination of outcomes and experiences of fathers participating in PYD and a systematic exploration of facilitation quality and differences in youth experiences with the new approach.

Several lessons emerged that highlight considerations for other practitioners or researchers that are planning to incorporate positive youth development strategies into their programs or transitioning to a more structured or prescribed program:

1. **Allow for more flexibility in program implementation** As written, the PYD model was highly structured and prescribed. Staff in multiple sites noted that the prescribed content in PYD did not always meet the specific needs of the young women they served. They suggested that greater discretion in allowing them to tailor or supplement activities would be helpful. For instance, some case managers said it was important to build in time for new mothers to talk about their child’s development and to build rapport before diving into structured activities. Staff also emphasized that each mother is different in terms of where she is starting and how she navigates the program. Some need more time and support and others need less. Program developers and trainers may wish to incorporate guidance on how and where supervisors and case managers can use their discretion on the best approach for each youth. For example, being flexible about the number of required visits each month would allow staff to invest time and resources in the mothers who have greater needs, closer to and in keeping with the original AFLP design.
2. **Strengthen training with field experience** The shift to PYD was a significant change for the case managers and supervisors. Case managers said the training did not fully prepare them to implement the new model. Particularly because the transition to PYD happened quickly in some sites, staff believed they needed more time to acclimate to the new model and practice the methods before they could begin serving young women using the PYD approach. Practitioners considering a shift to similar program models (particularly those that are more prescribed or use methods like motivational interviewing) may wish to provide in-depth preservice trainings for staff that directly address real-world implementation of the program. For instance, PYD case managers said it would be helpful for trainings to include more practical input from a case manager who could explain the different PYD requirements and components and how to use the new PYD methods and activities in practice. Staff suggested incorporating time to shadow and practice the methods, like motivational interviewing, in the field. PYD supervisors likewise suggested having trainings specifically for supervisors, in which they can learn how to best support their case managers in implementation and can learn from each other in interactive sessions.

## Footnotes

1 For two sites that offered both programs, the study team individually randomized new participants to either AFLP or PYD. At the remaining sites, the study team randomized the site to either AFLP or PYD.

2 Although the programs are open to fathers, only a small number of fathers participated, and they are not included in the study sample.

3 Impact findings based on the follow-up surveys will be presented in two separate forthcoming reports from OPA, including a report on the one-year impacts (Dragoset et al, 2020).

4 Both individuals that collected data were female. One was a researcher with a master’s degree in public health and over 10 years of experience with collecting qualitative data. The other team member was a research analyst with a master’s degree in public policy with 3 years of experience collecting qualitative data. Neither member of the team had a prior relationship with the staff or youth respondents.

5 Sixty-six of the expected 67 staff members completed the survey.

6 The staff survey was piloted formally with case managers in early sites. Interview guides were adjusted based on initial discussions with three sites who implemented early.

7 The team completed interviews with all expected respondents in each site, except for one supervisor in one site who was unable to participate because of staff turnover.

8 Focus group participants for AFLP and PYD had to have been enrolled in the program for at least three months and completed their first visit. The team attempted to engage with a wide variety of participants, but participation was voluntary and based on convenience and the participating young women’s availability. Focus group data do not include the perspectives of youth that dropped out of the programs or chose not to participate.

9 The first preservice trainings were four days long. However, after the first set of trainings that took place in 2015, MCAH streamlined the trainings to between two and three days for additional sites. Staff who attended the later trainings also received part of the motivational interview training online.

10 Trained study team members observed 42 individual visits (16 visits with AFLP participants and 26 visits with PYD participants) across the participating sites.
